# Microneedling Versus Chemical Peels for Atrophic Acne Scars: A Systematic Review and Meta-Analysis

**DOI:** 10.7759/cureus.108622

**Published:** 2026-05-11

**Authors:** Ifunanya Agu-Jefferson, Tsedena T Gebregiorgis, Lillian Ozumba, Osagioduwa Mike Atoe-Imagbe, Omotoyosi N Ilesanmi

**Affiliations:** 1 Acute/General Medicine, West Cumberland Hospital, Whitehaven, GBR; 2 General Internal Medicine, West Cumberland Hospital, Whitehaven, GBR; 3 Internal Medicine, West Cumberland Hospital, Whitehaven, GBR; 4 Dermatology, University of Ilorin Teaching Hospital, Ilorin, NGA

**Keywords:** acne scars, atrophic acne scars, chemical peels, dermapens, dermarollers, meta-analysis, microneedling, post-acne atrophic scars, systematic review

## Abstract

Atrophic acne scarring is a common complication of acne vulgaris and is associated with a significant psychosocial burden. Among the various treatment options available, microneedling and chemical peels are widely used due to their accessibility and favourable safety profiles. However, uncertainty remains regarding their comparative effectiveness and safety. Hence, the aim of this review is to compare the effectiveness of microneedling versus chemical peels in improving the severity of atrophic acne scars and to evaluate patient satisfaction and adverse events associated with these interventions.

Randomized and non-randomized clinical trials were included. Outcomes were categorized as clinically significant improvement (≥50% improvement), any improvement (≥1 grade improvement), and continuous measures of scar severity. Dichotomous outcomes were pooled using risk ratios (RR), while continuous outcomes were analyzed using standardized mean differences (SMD). Outcomes that could not be quantitatively synthesized were analyzed narratively.

A total of 11 studies comprising 713 participants were included. Meta-analysis of clinically significant improvement (five studies; n = 215) showed no significant difference between microneedling and chemical peels (RR = 0.97; 95% CI: 0.82-1.15; p = 0.74; I² = 0%). However, microneedling was associated with a significantly higher likelihood of achieving any improvement (four studies; n = 264; RR = 1.79; 95% CI: 1.37-2.34; p < 0.0001; I² = 0%). Analysis of continuous outcomes (four studies; n = 211) showed no significant difference, with a trend favouring chemical peels (SMD = −0.27; 95% CI: −0.91 to 0.38; p = 0.41; I² = 80%). Findings on patient satisfaction were inconsistent, while adverse events varied between interventions, with microneedling associated with greater procedural pain and chemical peels associated with burning sensations and exfoliation.

Microneedling and chemical peels appear to be broadly comparable in the treatment of atrophic acne scars, with no clear evidence of superiority. While microneedling may be more effective in achieving minimal improvement, and chemical peels may show advantages in certain continuous measures, these differences are not consistent across outcomes. Treatment decisions should therefore be individualized. Further high-quality studies with standardized outcome measures are needed to strengthen the evidence base.

## Introduction and background

Atrophic acne scarring is a common sequela of acne vulgaris and represents a significant clinical and psychosocial burden, often leading to reduced quality of life, impaired self-esteem, and long-term cosmetic concerns [1-3]. The management of acne scars remains challenging due to their heterogeneous presentation and the variable response to available treatment modalities.

A wide range of procedural interventions has been developed to improve acne scars, among which microneedling and chemical peeling are widely used due to their relative accessibility, cost-effectiveness, and favourable safety profiles [2,3]. Microneedling induces dermal remodeling through controlled micro-injury, stimulating collagen production and promoting skin regeneration [4,5]. In contrast, chemical peels, including glycolic acid, salicylic acid, trichloroacetic acid (TCA), and combination peels, promote skin renewal through controlled chemical exfoliation and dermal stimulation [1].

Despite their widespread clinical use, uncertainty remains regarding their comparative effectiveness, optimal selection based on scar characteristics, and safety outcomes across diverse patient populations and skin types. Existing reviews [5-10] have largely focused on individual treatment modalities or compared these interventions with other procedures, such as laser therapy, rather than providing a direct head-to-head synthesis. Consequently, there remains a lack of consolidated evidence to guide clinicians in choosing between these commonly used approaches.

Therefore, this systematic review and meta-analysis aims to directly compare microneedling and chemical peel interventions in the treatment of atrophic acne scars. The primary objective is to evaluate their effectiveness in improving scar severity, while secondary objectives include the comparison of patient satisfaction and associated adverse events.

## Review

Methodology

This review was conducted and reported following the guidelines of the Preferred Reporting Items for Systematic Reviews and Meta-Analyses (PRISMA) statement, and the protocol was registered at the International Prospective Register of Systematic Reviews (PROSPERO) (registration ID number: CRD420261304429).

Measured outcomes

The primary outcome was the effectiveness of microneedling compared to chemical peels in improving the severity of atrophic acne scars, while secondary outcomes included patient satisfaction and adverse events.

Given the variability in outcome reporting across studies, effectiveness outcomes were categorized into three main groups to facilitate appropriate synthesis. The first category comprised clinically significant improvement, defined as a ≥50% reduction in acne scar severity or equivalent thresholds, including upper quartile improvement or "good/excellent" response as reported in individual studies [11,12]. The second category included any improvement, defined as at least a one-grade improvement or equivalent categorical change in scar severity [11]. The third category consisted of continuous measures of acne scar severity, including post-acne scar scores and the Goodman and Baron grading system reported either as continuous variables or as ordinal categories [13]. For studies reporting ordinal Goodman and Baron grades, numerical values were assigned to categories to approximate continuous data, enabling inclusion in quantitative synthesis. Where multiple time points were reported, the most clinically relevant or final follow-up measurements were selected for analysis.

Eligibility criteria

The eligibility criteria were defined based on the PICOS (Population, Intervention, Comparator, Outcome, and Study design) strategy. The population included all individuals with clinically diagnosed atrophic acne scars (including rolling, boxcar, and icepick), regardless of age, sex, ethnicity, or skin phototype. The included intervention is specifically all forms of microneedling including dermaroller or automated microneedling devices, any needle length, treatment protocol, or number of sessions, but delivered as monotherapy and not in combination with any other treatment modality. The comparator comprised chemical peel treatments which are also delivered as monotherapy, including but not limited to glycolic acid, salicylic acid, TCA, Jessner's solution, and combination chemical peels. The outcome was as already defined in the previous subsection. The included study designs were randomized controlled trials (RCTs) and non-RCTs. Non-comparative studies including case reports and case series were excluded. Non-empirical studies including reviews, commentaries, and editorial letters were also excluded.

No restrictions were applied to the year of publication of papers, the setting of the included studies, or the language of the publications. Plans were made to translate papers written in a non-English language where necessary. No restriction was also placed on the follow-up duration of the interventions. To ensure the recruitment of studies with a good level of scientific methods, only studies published in peer-reviewed journals were recruited and reviewed; dissertations, grey literature, and books were excluded. Furthermore, to ensure uniformity in the assessment of methodological quality, only full-text articles were retrieved.

Search strategy

Information was sought from scientific databases including MEDLINE, Embase, Scopus, the Cumulative Index to Nursing and Allied Health Literature (CINAHL), and Cochrane Central Register of Controlled Trials (CENTRAL).

The search strategy employed in the literature search was as follows: (microneedle* OR dermaroller OR dermapen OR "percutaneous collagen induction") AND ("chemical peel*" OR chemexfoliation OR glycolic OR salicylic OR "trichloroacetic acid" OR TCA OR Jessner) AND (acne AND scar*).

The search strategy was tailored for each database, and subject headlines for keywords corresponding to microneedling, chemical peels, and acne were also sought and combined into the search strategy using Boolean operators. The detailed search strategy for each database is presented in Appendix A.

Searching and selection of studies

All retrieved records were imported into Mendeley®, a reference management software developed by Elsevier (Amsterdam, Netherlands), and duplicates were removed using the tool.

Three reviewers (IA, TG, and LO) screened the titles and abstracts for relevancy, and full texts were sought for after this. Conference papers and papers with no open access to full texts were excluded. Full-text eligibility screening was conducted on Microsoft Excel (Microsoft Corp., Redmond, WA, USA) by two independent reviewers (IA and TG), and disagreements were resolved by discussion.

A PRISMA flowchart was used to document the whole searching and screening process and was generated using the online Shiny app tool (Posit, Boston, MA, USA) [14].

Assessment of risk of bias

Risk of bias assessments were performed by two reviewers (IA and TG) and cross-checked by another reviewer (OI). This was performed with two different tools according to the type of study. For the randomized studies, the Cochrane Risk of Bias Tool (RoB 2.0) [15] was used, while for the non-randomized trial, the Risk of Bias in Non-randomized Studies of Interventions (ROBINS-I) tool [16] was used. No study was excluded for its rated risk of bias; however, the methodology quality was considered during data synthesis and results interpretation.

Data extraction

Data were extracted independently by one reviewer (IA) and cross-checked by two reviewers (LO and OA). The extraction was done using a standardized data extraction form developed for this review. Any discrepancies between reviewers were resolved through discussion.

The following information was extracted from each included study: author and year of publication, country of study, study aim, study design, participant characteristics (sample size, age range, sex distribution, skin type, and type of acne scars), intervention details for microneedling (device type, needle depth, and number of treatment sessions), comparator details for chemical peels (type of peeling agent, concentration, and number of sessions), and outcome data (the acne scar grading or assessment tools used, reported changes in scar severity, patient-reported outcomes including satisfaction, and adverse effects associated with treatment). Where multiple outcome measures or time points were reported, data were extracted for all relevant measures, and the most clinically appropriate or commonly reported time points were selected for synthesis.

Data synthesis

Given that acne scar improvement was measured using a variety of categorical and continuous scales, a structured approach was adopted to ensure that only clinically and methodologically comparable outcomes were quantitatively pooled. To achieve this, outcomes were grouped into conceptually similar categories based on how improvement was defined and reported.

The first, and primary outcome (clinically significant improvement of ≥50%), was pooled using risk ratios (RRs) with 95% confidence intervals (CI). Sequentially, studies reporting any degree of improvement, such as ≥1 grade change on ordinal scales, were analyzed separately as a secondary outcome. This outcome reflects a lower threshold of clinical response and was not combined with a ≥50% improvement outcome in order to avoid misclassification and preserve interpretability. It was also pooled using RRs.

For studies reporting acne scar severity as continuous or quasi-continuous variables, a separate synthesis was performed. These included post-acne scar scores, continuous Goodman and Baron scores, and studies reporting Goodman and Baron grades as distributions. In the latter case, ordinal grades were converted into numerical values (grades 1-4) to approximate continuous data, enabling inclusion in the quantitative synthesis. This approach assumes equal intervals between ordinal categories, which may not strictly reflect the underlying clinical scale; however, it is a commonly accepted method when pooling outcomes measured using different instruments that assess the same construct, particularly when standardized mean differences (SMDs) are used [17,18]. Post-intervention values were used for continuous outcome meta-analysis, as change score standard deviations were not consistently reported. This approach is consistent with methodological guidance indicating that, in randomized studies, post-treatment comparisons provide valid estimates of intervention effects [17].

Studies that reported only pre- and post-intervention distributions of ordinal severity grades without providing individual-level change data were not converted into dichotomous responder outcomes, as transitions between severity categories could not be reliably inferred. Such studies were only included in the continuous outcome analysis.

Meta-analyses were conducted when at least two studies reported sufficiently comparable outcomes. Given the anticipated clinical and methodological heterogeneity arising from differences in treatment protocols, a random-effects model was used for all analyses. This approach accounts for both within-study and between-study variability and is recommended when heterogeneity is expected [19].

Statistical heterogeneity was assessed using the I² statistic and Cochran's Q test. Thresholds for interpretation followed conventional guidance, with values of approximately 25%, 50%, and 75% representing low, moderate, and high heterogeneity, respectively, although these were interpreted in the context of clinical diversity among studies [17].

Originally planned subgroup analyses included skin type, scar type, scar severity, chemical peel type, microneedling technique, and duration of follow-up. However, this could not be conducted in all the meta-analyses because of the limited number of studies per group. Nevertheless, sensitivity analyses were conducted by excluding studies at high risk of bias. All meta-analyses were conducted using Cochrane's Review Manager software, version 5.4 (The Cochrane Collaboration, London, England, UK).

In addition to quantitative synthesis, a narrative approach was used for outcomes that could not be meaningfully pooled due to heterogeneity in reporting formats, outcome definitions, or insufficient data. This applied particularly to patient satisfaction and adverse events, where studies used diverse categorical scales and inconsistent reporting methods. This approach is consistent with established guidance for the synthesis of heterogeneous data in systematic reviews [20].

The certainty of evidence for each outcome was assessed using the GRADE (Grading of Recommendations, Assessment, Development and Evaluation) approach. This involved evaluation of risk of bias, inconsistency, indirectness, imprecision, and publication bias. The certainty of evidence was classified as high, moderate, low, or very low.

Results of the search and screening process

**Figure 1:**
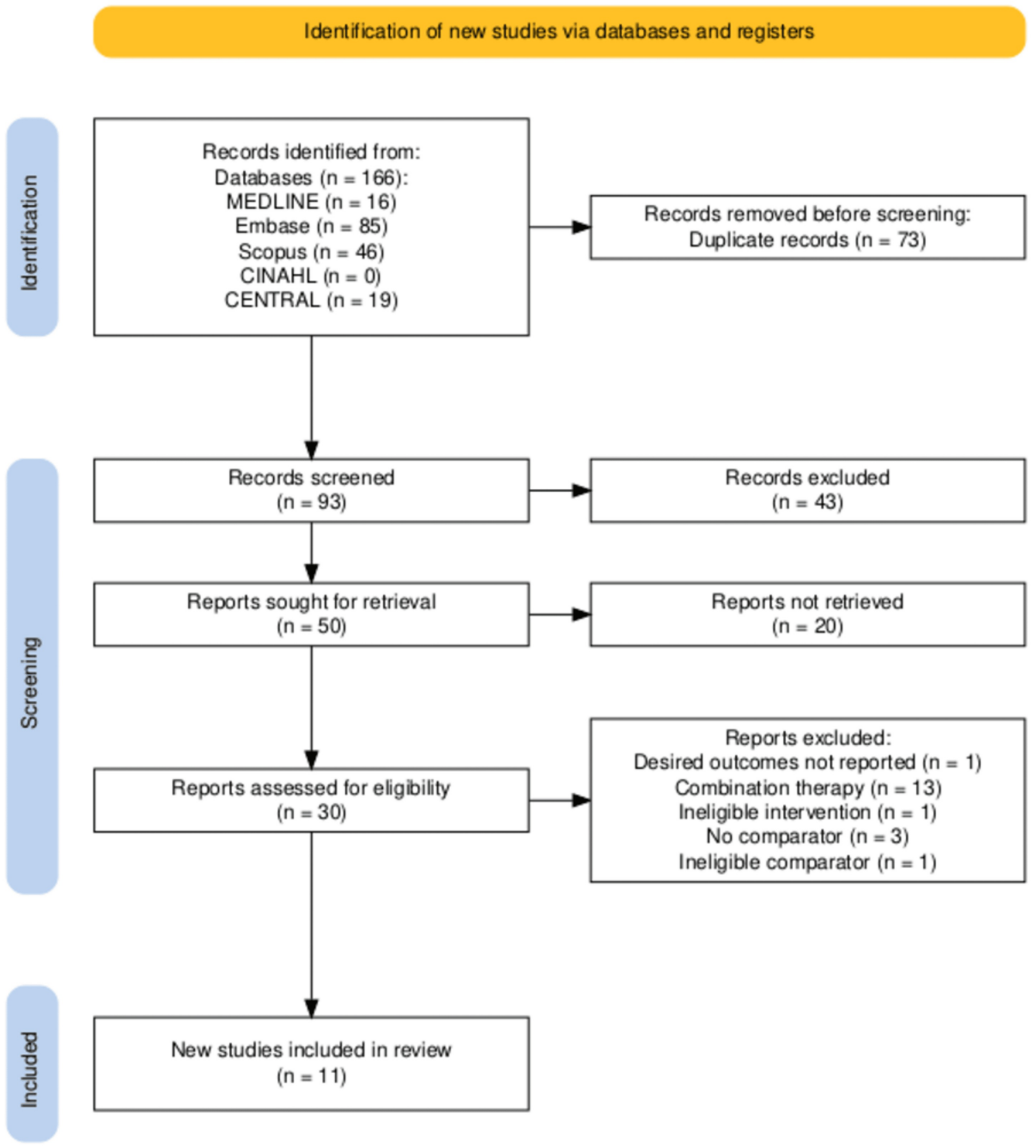
PRISMA flowchart for this review PRISMA: Preferred Reporting Items for Systematic Reviews and Meta-Analyses; CINAHL: Cumulative Index to Nursing and Allied Health Literature; CENTRAL: Cochrane Central Register of Controlled Trials

The database search identified a total of 166 records. No additional records were identified through other sources. After the removal of duplicates, 93 unique records remained and were screened based on titles and abstracts. Of these, 43 records were excluded for not meeting the inclusion criteria. A total of 50 records were initially considered for full-text assessment; however, the full texts of 20 articles could not be retrieved despite attempts to access them. Consequently, 30 full-text articles were assessed for eligibility. Of these, 19 studies were excluded for various reasons. The details of the eligibility screening decision matrix are provided in Appendix B. A total of 11 studies [21-31] met the inclusion criteria and were included in the final analysis. The study selection process is illustrated in Figure 1.

Risk of bias assessment

The methodological quality of the included studies was assessed using the Cochrane Collaboration RoB 2.0 tool for RCTs [15] and the ROBINS-I tool for the non-randomized study [16].

Among the 10 RCTs, only one study was judged to be at low risk of bias [27], while seven studies were assessed as having some concerns [22,24-26,28,30,31] and two studies were judged to be at high risk of bias [21,29]. The most common sources of bias arose from limitations in the randomization process and lack of blinding, particularly given the procedural nature of the interventions, which made participant and assessor blinding challenging. Selective reporting was also identified as a concern in some studies, where outcomes described in the methods were incompletely reported in the results. Additionally, the use of subjective scar assessment scales contributed to measurement bias, as outcome evaluation often relied on clinician judgment without clear evidence of blinding or standardized assessment procedures.

The single non-randomized comparative study [23] was assessed as having a moderate risk of bias using the ROBINS-I tool, primarily due to potential confounding and limitations inherent in non-randomized study designs.

**Figure 2:**
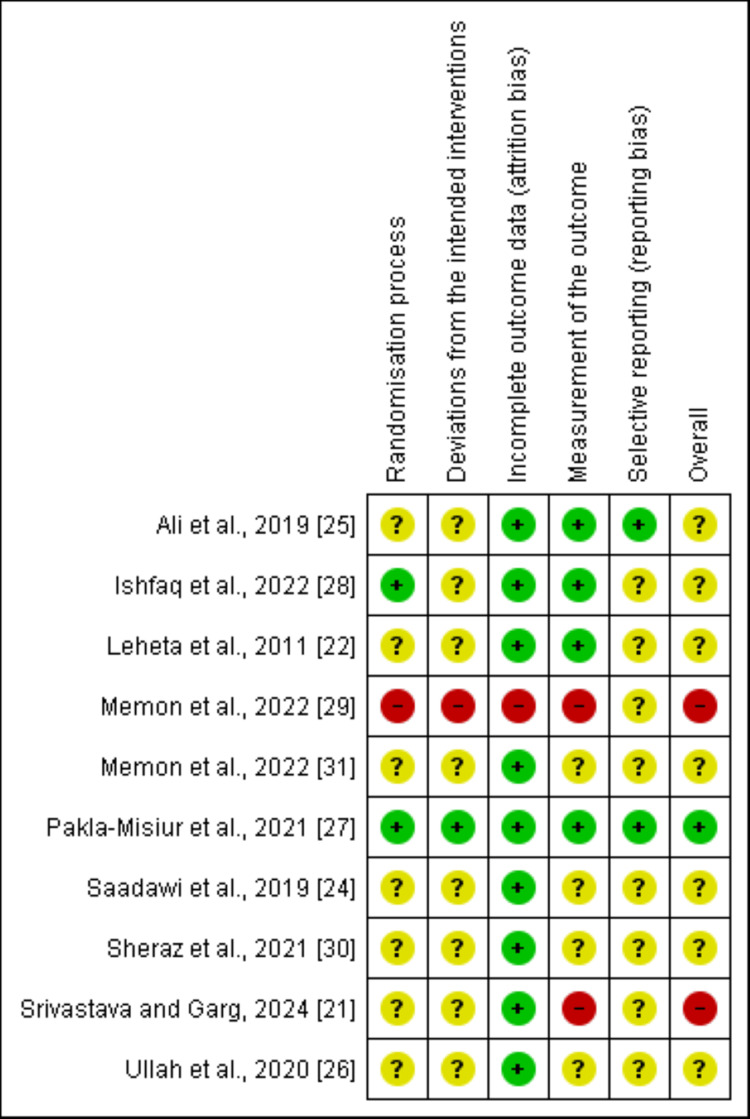
Risk of bias summary for the randomized controlled trials

**Figure 3:**
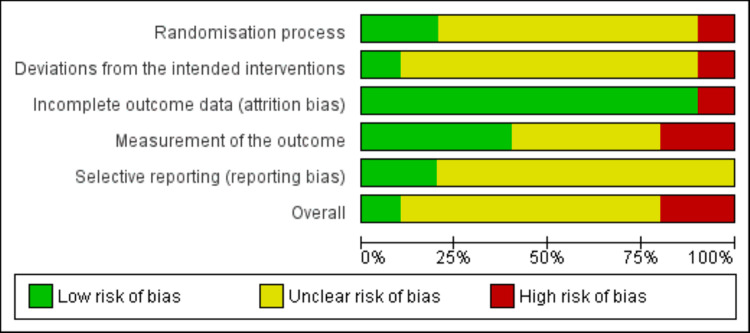
Risk of bias graph for the randomized controlled trials

A summary of the risk of bias findings for the RCTs is presented in Figure 2 and Figure 3, and the detailed decision matrix for the assessments is reported in Appendix C. No graphical representation was generated for the non-randomized study.

Characteristics of the included studies 

A total of 11 studies were included in this review, comprising 10 RCTs and one non-randomized trial. The studies were conducted across multiple countries, with the majority originating from Pakistan (n = 5) [26,28-31] and Egypt (n = 3) [22,24,25], while the remaining were conducted in India (n = 2) [21,23] and Poland (n = 1) [27].

The included studies involved a total of 713 participants, with 357 allocated to microneedling interventions and 356 to chemical peel comparators. Participants were predominantly young to middle-aged individuals, with reported ages ranging from 15 to 50 years. Sex distribution showed a higher proportion of female participants overall (413 females and 263 males); however, one study [25] did not report sex distribution.

Skin type was inconsistently reported, with only five studies [21,22,24,28,31] providing information based on Fitzpatrick classification, ranging from types II to VI. Similarly, acne scar types were not uniformly described; three studies did not report scar morphology, while the remaining studies included a mix of rolling, boxcar, and icepick scars.

Microneedling interventions were delivered using either dermarollers (n = 6) [21-23,26,30,31] or automated devices such as dermapens (n = 4) [24,25,27,29], while one study [28] did not specify the device used. A variety of chemical peeling agents were used as comparators, including glycolic acid (n = 4) [24,28,29,31], TCA (n = 4) [22,23,26,30], Jessner's solution (n = 1) [25], salicylic acid (n = 1) [21], and combination peels (n = 1) [27]. Reported concentrations ranged widely from 20% to 100%, and, similar to microneedling, the number of treatment sessions ranged from four to 12.

There was considerable heterogeneity in outcome assessment methods across studies. The most commonly used approach was quartile-based grading of improvement (n = 5) [22-26], followed by assessment of improvement defined as at least one grade change (n = 4) [21,27,28,31].

A detailed summary of the characteristics of the included studies is presented in Table 1.

**Table 1:** Extracted data from the studies included in this review RCT: randomized controlled trial; NS: not specified; mm: millimeters; TCA: trichloroacetic acid; CROSS: chemical reconstruction of skin scars

Author and year	Country	Study design and sample size	Participant characteristics	Intervention (microneedling) details	Comparator (chemical peel) details	Scar grading tool used	Result of scar changes (microneedling group)	Result of scar changes (chemical peel group)	Satisfaction outcomes (microneedling group)	Satisfaction outcomes (chemical peel group)	Adverse events (microneedling group)	Adverse events (chemical peel group)
Ali et al., 2019 [25]	Egypt	RCT: n = 40 (20 each in both groups)	Age range: NS. Sex: NS. Skin type: NS. Scar type: boxcar scars, rolling scars, icepick scars	Device type: Dermapen needle. Depth: 2.5 mm. Number of sessions: 8	Agent: Jessner's solution. Concentration: lactic acid (85%) 14 g, 14 g salicylic acid, resorcinol 14 g, and ethanol to 100 ml. Applied in multiple layers of 4-7 coats. Number of sessions: 8	Goodman and Baron clinical efficacy: very significant = >75-100%; marked = >50-75%; moderate = >25-50%; mild = <25%	Before treatment: Grade IV = four patients (20%). Grade III = 10 patients (50%). Grade II = six patients (30%)	Before treatment: Grade IV = 10 patients (50%). Grade III = 8 patients (40%). Grade II = 2 patients (10%)	-	-	Pain: mild = 0, moderate = 4 (20%), severe = 4 (20%). Erythema = 4 (20%). Exfoliation = 0	Pain: mild = 0, moderate = 0, severe = 0. Erythema = 3 (15%). Exfoliation = 4 (20%)
							After treatment: Grade IV = four patients (20%). Grade III = four patients (20%). Grade II = six patients (30%). Grade I = six patients (30%)	After treatment: Grade IV = nine patients (45%). Grade III = 9 patients (45%). Grade II = 2 patients (30%)				
							Improvement level: mild = four patients (20%); moderate = 10 patients (50%); marked = four patients (20%); and very significant = two patients (10%)	Improvement level: mild = eight patients (40%); moderate = 7 patients (35%); marked = four patients (20%); and very significant = one patients (5%)				
Ishfaq et al., 2022 [28]	Pakistan	RCT: n = 60 (30 in the microneedling group and 30 in the chemical peel group)	Age range: 15-50 years. Sex: 41 females and 19 males. Skin type: Fitzpatrick skin phototypes 4-6. Scar type: icepick, rolling, and boxcar scars	Device type: NS. Needle depth: NS. Number of sessions: 12	Agent: glycolic acid. Concentration: 35%. Number of sessions: 12	Goodman and Baron efficacy defined as an improvement greater than one grade from baseline	Yes = 22 (73.33%). No = 8 (26.67%)	Yes = 10 (33.33%). No = 20 (66.77%)	-	-	-	-
Leheta et al., 2011 [22]	Egypt	RCT: n = 27 (15 in the microneedling group and 12 in the chemical peel group)	Age range: 19-42 years. Sex: 16 men and 14 women. Skin type: Fitzpatrick 2, 3, 4. Scar type: icepick, rolling, and boxcar scars	Device type: dermaroller needle. Depth: 1.5 mm. Number of sessions: 4	Agent: TCA. Concentration: 100%. Number of sessions: 4	Disease severity score (post-acne scar score)	Mean post-acne score before treatment = 74.8 ± 35.6	Mean post-acne score before treatment = 79.6 ± 32.8	-	-	Mean pain score (0-9) = 5.4 ± 1.9. Mean duration of erythema and oedema = 3.0 ± 0.8 days	Mean pain score (0-9) = 3.8 ± 1.6. Mean duration of erythema and oedema = 15.9 ± 4.3 days
							Mean post-acne score after treatment = 25.2 ± 23.0	Mean post-acne score after treatment = 19.7 ± 13.7				
						Response rate using quartile grading scale: slight or no improvement = <25%; mild = 25-49%; moderate = 50-74%; significant = ≥75%	Percentage improvement after treatment = 68.3 ± 19.3	Percentage improvement after treatment = 75.3 ± 9.4				
							Improvement level: slight or no improvement = 1 (6.7%); mild = 2 (13.3); moderate = 5 (33.3%); significant = 7 (46.7%)	Improvement level: slight or no improvement = 0 (0%); mild = 1 (8.3%); moderate = 3 (25%); significant = 8 (66.7%)				
Memon et al., 2022 [31]	Pakistan	RCT: n = 60 (30 each in both groups)	Age range: 20-40 years. Sex: 41 females and 19 males. Skin type: Fitzpatrick 4, 5, 6. Scar type: icepick, rolling, and boxcar scars	Device type: dermaroller needle. Depth: 0.5 mm. Number of sessions: every 2 weeks for 5 months	Agent: glycolic acid. Concentration: 30%. Number of sessions: every 2 weeks for 5 months	Goodman and Baron efficacy defined as an improvement greater than one grade from baseline	Yes = 21 (70%). No = 9 (30%)	Yes = 11 (36.7%). No = 20 (63.3%)	-	-	-	-
Memon et al., 2022 [29]	Pakistan	RCT: n = 80 (40 each in both groups)	Age range: 18-41 years. Sex: 35 males and 45 females. Skin type: NS. Scar type: icepick, rolling, and boxcar scars	Device type: Dermapen needle. Depth: NS. Number of sessions: 6	Agent: glycolic acid. Concentration: 35%. Number of sessions: 6	Standard global scar grading system	Improvement level: no improvement (15%); mild (42%); good (43%); very good (0%)	Improvement level: no improvement (31%); mild (50%); good (19%); very good (0%)	-	-	-	-
Pakla-Misiur et al., 2021 [27]	Poland	RCT: n = 81 (40 in the microneedling group and 41 in the chemical peel group)	Age range: 18-45 years. Sex: 18 males and 63 females. Skin type: NS. Scar type: NS	Device type: Dermapen needle. Depth: NS. Number of sessions: 4	Agent: TCA, kojic acid, hydrogen peroxide. Concentration: TCA (33%), kojic (5%), hydrogen peroxide (5%). Number of sessions: 4	Goodman and Baron efficacy defined as an improvement greater than one grade from baseline	Mean Goodman and Baron score before treatment = 2.88 ± 0.98	Mean Goodman and Baron score before treatment = 2.50 ± 1.03	-	-	-	-
							Mean Goodman and Baron score after treatment = 2.55 ± 0.92	Mean Goodman and Baron score after treatment = 2.28 ± 0.95				
							Effective: Yes = 13 (32.5%). No = 27 (67.5%)	Effective: Yes = 10 (24.4%). No = 31 (75.6%)				
Puri, 2015 [23]	India	Non-randomized trial: n = 30 (15 each in both groups)	Age range: 11-40 years. Sex: 24 females and 6 males. Skin type: NS. Scar type: NS	Device type: dermaroller needle. Depth: 1.5-2.5 mm. Number of sessions: 4	Agent: TCA. Concentration: 70%. Number of sessions: 4	Non-specific response rate = poor (<30%); fair (30-50%); good (50-70%); and excellent (>70%)	Improvement level: mild = 3 patients (20%); moderate = 6 patients (40%); and marked = 6 patients (40%)	Improvement level: Mild = 2 patients (13.3%); moderate = 4 patients (26.7%); and marked = 9 patients (60%)	-	-	Pain = 1 (6.7%). Erythema = 3 (20%). Hyperpigmentation = 0	Pain = 0. Erythema = 0. Hyperpigmentation = 2 (13.3%)
Saadawi et al., 2019 [24]	Egypt	RCT: n = 20 (10 each in both groups)	Age range: 19-45 years. Sex: 8 men and 12 women. Skin type: Fitzpatrick 2, 3, 4. Scar type: icepick, rolling, and boxcar scars	Device type: Dermapen needle. Depth: NS. Number of sessions: 6	Agent: glycolic acid. Concentration: 35%. Number of sessions: 6	Goodman and Baron and quartile grading scale: poor or no improvement (<25%); mild (25-49%); good (50-74%); and very good improvement (≥75%)	Improvement level: no improvement = 2 (20%); mild = 4 (40%); good = 4 (40%); very good = 0 (0%)	Improvement level: no improvement = 3 (30%); mild = 5 (50%); good = 2 (20%); very good = 0 (0%)	Mild = 2 (20%); good = 6 (60%); and very good = 2 (20%)	Mild = 6 (60%); good = 3 (30%); and very good = 1 (10%)	None = 1 (10%). Erythema = 3 (30%). Pain = 6 (60%). Acne flare = 0 (0%). Burning sensation = 0 (0%)	None = 2 (20%). Erythema = 0 (0%). Pain = 0 (0%). Acne flare = 1 (10%). Burning sensation = 7 (70%)
Sheraz et al., 2021 [30]	Pakistan	RCT: n = 154 (77 each in both groups)	Age range: 18-40 years. Sex: 90 males and 64 females. Skin type: NS. Scar type: icepick, rolling, and boxcar scars	Device type: dermaroller needle. Depth: NS. Number of sessions: 4	Agent: TCA. Concentration: 70%. Number of sessions: 4	Clinical grading using a non-specified system, in addition to the patients' satisfaction level	Effective: Yes = 31 (40.3%). No = 46 (59.7%)	Effective: Yes = 46 (59.7%). No = 31 (40.3%)	-	-	-	-
Srivastava and Garg, 2024 [21]	India	RCT: n = 63 (31 in the microneedling group and 32 in the chemical peel group)	Age range: 18-45 years. Sex: 41 females and 22 males. Skin type: Fitzpatrick skin types 4 and 5. Scar type: NS	Device type: dermaroller needle. Depth: 1.5 × 0.25 mm. Number of sessions: 4	Agent: salicylic acid. Concentration: 20%. Number of sessions: 4	Goodman and Baron	Before treatment: Grade IV = seven patients (22.6%); Grade III = 15 patients (48.4%); Grade II = eight patients (25.8%); Grade 1 = 1 patient (3.2%).	Before treatment: Grade IV = four patients (12.5%); Grade III = 14 patients (43.8%); Grade II = 11 patients (34.4%); Grade I = 3 patients (9.4%).	Poor = 19 (59.4%); good = 8 (25%); and excellent = 5 (15.6%)	Poor = 7 (22.6%); good = 9 (29%); and excellent =15 (48.4%)	Oedema = 8 (25.8%). Erythema = 6 (19.4%). Hyperpigmentation = 6 (19.4%). Secondary infection = 0 (0%)	Oedema = 0 (0%). Erythema = 10 (31.3%). Hyperpigmentation = 5 (15.6%). Secondary infection = 0 (0%)
						Improvement grading: excellent response = two or more grade improvements. Good response = single grade improvement. Poor response = same scar grading after treatment	After treatment: Grade IV = four patients (12.9%); Grade III = two patients (6.5%); Grade II = five patients (16.1%); Grade I = 19 patients (61.3%); complete resolution = one patient (3.2%)	After treatment: Grade IV = four patients (12.5%); Grade III = 12 patients (37.5%); Grade II = 5 patients (15.6%); Grade I = 3 patients (9.4%); complete resolution = 8 patients (25%)				
						Patient satisfaction (graded on a 10-point scale): >7 = excellent response; 4 to 7 = good response; <4 = poor response	Improvement level: poor = 8 patients (25.8%); good = 9 patients (29%); and excellent = 14 patients (45.2%)	Improvement level: poor = 18 patients (56.3%); good = 8 patients (25%); and excellent = 6 patients (18.8%)				
Ullah et al., 2020 [26]	Pakistan	RCT: n = 98 (49 each in both groups)	Age range: 20-39 years. Sex: 30 males and 68 females. Skin type: NS. Scar type: icepick, rolling, and boxcar scars	Device type: dermaroller needle depth: 1.5-2.5 mm. Number of sessions: 4	Agent: TCA. Concentration: 100%. Number of sessions: 4	Quartile grading scale: poor = <25%; fair = 26-50%; good = 51-75%; excellent = >75%	Improvement level: poor = 7 (14.3%); fair = 9 (18.4%); good = 18 (36.7%); excellent = 15 (30.6%)	Improvement level: poor = 5 (10.2%); fair = 13 (26.5%); good = 16 (32.7%); excellent = 15 (30.6%)	-	-	-	-

Effect of microneedling versus chemical peels on acne scar severity

Clinically Significant Improvement (≥50% Improvement) (Primary Outcome)

**Figure 4:**
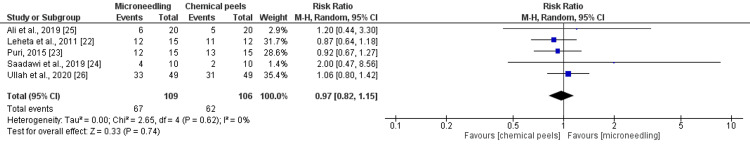
Forest plot of comparison of clinically significant improvement (≥50% improvement) between microneedling and chemical peels

Five studies [22-26] involving a total of 215 participants were included in the meta-analysis assessing clinically significant improvement. The pooled analysis demonstrated no statistically significant difference between microneedling and chemical peel interventions (RR = 0.97; 95% CI: 0.82-1.15; p = 0.74). There was no evidence of statistical heterogeneity among the included studies (I² = 0%), and a visual inspection of the forest plot did not suggest the presence of outlier studies or substantial variation in effect direction. Given that all included studies were assessed as having a moderate risk of bias, sensitivity analysis based on study quality was not performed. The results of this analysis are presented in Figure 4.

Any Improvement (≥1 Grade Improvement)

Four studies [21,27,28,31] involving a total of 264 participants were included in the meta-analysis assessing any improvement in acne scar severity, defined as at least a one-grade improvement. The pooled analysis demonstrated that microneedling was associated with a significantly higher likelihood of improvement compared to chemical peels (RR = 1.79; 95% CI: 1.37-2.34; p < 0.0001). There was no evidence of statistical heterogeneity among the included studies (I² = 0%), and a visual inspection of the forest plot did not reveal any outlier studies or substantial variation in effect estimates.

**Figure 5:**
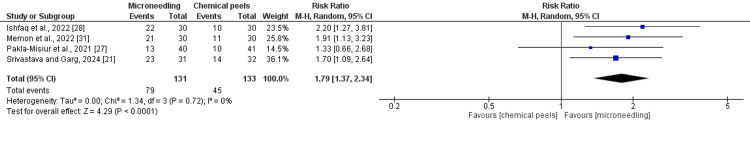
Forest plot of comparison of any improvement (≥1 grade improvement) between microneedling and chemical peels

Sensitivity analysis was performed to assess the influence of study quality by excluding the study assessed as having a high risk of bias [21]. This did not materially alter the overall findings, with the results remaining statistically significant and in favour of microneedling. The results of this analysis are presented in Figure 5.

Changes in Acne Scar Severity (Continuous Outcomes)

Four studies [21,22,25,27] involving a total of 211 participants were included in the meta-analysis of continuous outcomes assessing changes in acne scar severity. The pooled analysis showed no statistically significant difference between microneedling and chemical peel interventions (SMD = −0.27; 95% CI: −0.91 to 0.38; p = 0.41). The direction of effect slightly favoured chemical peels; however, the magnitude of the effect was small and not statistically significant. Interpretation of this finding is further limited by substantial statistical heterogeneity among the included studies (I² = 80%). Despite this, visual inspection of the forest plot did not reveal any obvious outlier studies or marked inconsistency in effect direction.

**Figure 6:**
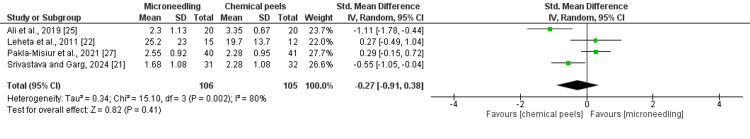
Forest plot of comparison of continuous outcomes between microneedling and chemical peels

Sensitivity analysis was conducted by excluding the study assessed as having a high risk of bias [21], and this did not materially alter the overall findings, with the results remaining non-significant. The results of this analysis are presented in Figure 6.

Patient satisfaction

Patient satisfaction was reported in two studies [21,24], with both using ordinal categorical scales to assess perceived treatment outcomes. Due to differences in the categorization and reporting of satisfaction levels, a quantitative synthesis was not performed, and findings are presented narratively.

In the study by Saadawi et al. [24], patient satisfaction favoured microneedling, with a higher proportion of participants reporting favourable outcomes. In the microneedling group, 80% of participants reported "good" or "very good" satisfaction, compared to 40% in the chemical peel group. Conversely, a greater proportion of patients in the chemical peel group reported only mild satisfaction (60% vs 20%).

In contrast, the study by Srivastava and Garg [21] reported higher satisfaction among participants treated with chemical peels. In this study, 48.4% of participants in the chemical peel group reported "excellent" outcomes compared to 15.6% in the microneedling group. Additionally, a higher proportion of participants in the microneedling group reported poor satisfaction (59.4% vs 22.6%).

Overall, the findings on patient satisfaction were inconsistent across studies, with one study favouring microneedling and the other favouring chemical peels. The limited number of studies and variation in outcome reporting preclude definitive conclusions regarding patient-reported satisfaction.

Adverse events

Adverse events were reported in five studies [21-25], with considerable variation in the type and reporting of outcomes. Due to this heterogeneity, a quantitative synthesis was not performed, and findings are presented narratively. Overall, both microneedling and chemical peel interventions were associated with generally mild and transient adverse effects, although the type and frequency of events differed between treatments.

Pain and erythema were the most commonly reported adverse events. In three studies [22,24,25], microneedling was associated with a higher frequency and intensity of procedural pain, with proportions ranging between 40% and 60% and above-average mean score for continuous scales, compared to zero proportion and below-average mean score for chemical peels. Erythema was reported variably in both groups across multiple studies [21-24], although its duration differed. Nevertheless, Leheta et al.'s [22] findings suggest that erythema resolved more quickly following microneedling (mean duration 3.0 ± 0.8 days) compared to chemical peels (15.9 ± 4.3 days).

Other adverse events varied by intervention. Chemical peels were more frequently associated with exfoliation and burning sensations [24,25]. Post-inflammatory hyperpigmentation was observed in both treatment groups, with no consistent pattern favouring either intervention [21,23]. Other less frequently reported adverse events included oedema and acne flare, with no clear differences between interventions [21,22,24]. No cases of secondary infection were reported in the included studies [21].

Certainty of evidence

The certainty of evidence for the assessed outcomes was evaluated using the GRADE approach. Overall, the certainty of evidence ranged from moderate to very low. Evidence for clinically significant improvement (≥50% improvement) was rated as low certainty due to concerns regarding risk of bias and imprecision. Evidence for any improvement (≥1 grade improvement) was rated as moderate certainty, with downgrading primarily due to risk of bias. Continuous outcomes assessing acne scar severity were rated as very low certainty due to substantial heterogeneity, imprecision, and methodological limitations. Similarly, the certainty of evidence for patient satisfaction and adverse events was rated as very low due to limited data and inconsistent reporting across studies. The details are provided in Table 2 below.

**Table 2:** Summary of findings and certainty of evidence (GRADE) GRADE: Grading of Recommendations, Assessment, Development and Evaluation

Outcome	No. of studies (participants)	Effect estimate	Certainty of evidence (GRADE)	Reasons for downgrading
Clinically significant improvement (≥50%)	5 (n = 215)	RR = 0.97 (0.82-1.15)	Low	Risk of bias, imprecision
Any improvement (≥1 grade)	4 (n = 264)	RR = 1.79 (1.37-2.34)	Moderate	Risk of bias
Continuous scar severity	4 (n = 211)	SMD = −0.27 (−0.91 to 0.38)	Very low	Risk of bias, inconsistency, imprecision
Patient satisfaction	2 studies	Narrative	Very low	Inconsistency, imprecision
Adverse events	5 studies	Narrative	Very low	Inconsistency, imprecision

Discussion

This systematic review and meta-analysis aimed to compare the effectiveness of microneedling and chemical peels in the management of atrophic acne scars while also evaluating patient satisfaction and adverse events. Overall, the findings suggest that both interventions are broadly comparable, with no consistent evidence supporting the superiority of either modality.

The primary outcome, defined as clinically significant improvement (≥50% reduction in acne scar severity), showed no statistically significant difference between microneedling and chemical peels. This aligns with the broader dermatologic literature, which suggests that multiple minimally invasive procedures can achieve comparable improvements in atrophic acne scars when applied appropriately [1,11]. These findings reinforce the concept that treatment efficacy is often influenced more by patient selection and technique than by the modality itself [12,32].

However, when a less stringent threshold of improvement (≥1 grade improvement) was applied, microneedling demonstrated a statistically significant advantage. This observation is consistent with studies suggesting that microneedling induces collagen remodeling through controlled dermal injury, leading to gradual but noticeable improvements in scar texture [33-35]. It is plausible that microneedling produces early or subtle improvements that are captured by less stringent outcome measures but may not translate into more substantial clinical change.

In contrast, the analysis of continuous outcomes suggested a non-significant trend favouring chemical peels. Although not statistically significant, this finding may reflect the mechanism of action of chemical peels, which promote controlled chemical exfoliation and dermal regeneration, potentially leading to more uniform improvements in skin texture over time [36,37]. Continuous scoring systems may be more sensitive to these incremental changes compared to categorical thresholds. The divergence between categorical and continuous outcomes highlights a well-recognized challenge in acne scar research which is the lack of standardized outcome measures [38].

Patient satisfaction findings were inconsistent across studies, with one favouring microneedling and the other favouring chemical peels. This variability is not unexpected, as patient-reported outcomes are influenced by multiple factors beyond objective clinical improvement, including pain, downtime, expectations, and cosmetic preferences. Previous studies have similarly reported discordance between clinician-assessed outcomes and patient satisfaction in aesthetic dermatology [39-41]. The limited reporting of satisfaction outcomes in the included studies further restricts meaningful interpretation.

Adverse event profiles differed between interventions. Microneedling was more frequently associated with procedural pain and transient erythema, while chemical peels were associated with burning sensations, exfoliation, and occasional post-inflammatory hyperpigmentation. These findings are consistent with known safety profiles of both treatments [34,37]. Notably, the shorter duration of erythema observed with microneedling in some studies suggests a potentially shorter recovery period, which may be clinically relevant when considering patient preference and return to normal activities.

The overall findings of this review are in keeping with existing evidence indicating that both microneedling and chemical peels are effective treatment modalities for atrophic acne scars, with no clear superiority of one over the other. Previous comparative and non-comparative studies have highlighted the importance of combining treatments or tailoring interventions based on scar type and patient characteristics to achieve optimal outcomes [42-48].

Several limitations should be considered when interpreting these findings. The relatively small number of included studies limits the robustness of the conclusions and precluded meaningful subgroup analyses. Clinical and methodological heterogeneity was substantial, particularly in terms of treatment protocols, outcome measures, and follow-up durations. Outcome reporting was inconsistent across studies, with a wide range of subjective and objective assessment tools used. The reliance on subjective scar grading scales introduces potential measurement bias, especially in the absence of blinded outcome assessment. Furthermore, the conversion of ordinal data into continuous measures, while methodologically acceptable, involves assumptions that may reduce precision. The inability to explore the effects of important variables such as scar type, scar severity, skin type, treatment parameters, and session frequency represents an additional limitation.

Despite these limitations, this review has notable strengths. A comprehensive and systematic approach was employed, with rigorous study selection, data extraction, and risk of bias assessment. The use of multiple analytical approaches, including both dichotomous and continuous outcomes, allowed for a more nuanced evaluation of treatment effects. Additionally, the inclusion of safety and patient-reported outcomes provides a broader clinical perspective.

From a clinical standpoint, the findings suggest that both microneedling and chemical peels are viable and comparable treatment options for atrophic acne scars. In the absence of clear superiority, treatment decisions should be individualized, taking into account patient preferences, tolerance for adverse effects, cost, and clinician expertise.

Future research should prioritize well-designed RCTs with standardized outcome measures and longer follow-up periods. Establishing consensus on clinically meaningful outcome thresholds and improving reporting of patient-centered outcomes will be essential to advancing the field and enabling more definitive comparisons.

## Conclusions

This systematic review and meta-analysis found that microneedling and chemical peels are broadly comparable in the management of atrophic acne scars, with no clear evidence supporting the superiority of either intervention in achieving clinically significant improvement. While microneedling was associated with a higher likelihood of achieving minimal improvement, and chemical peels showed a non-significant trend towards greater improvement in continuous measures of scar severity, these differences were not consistent across outcomes. Findings relating to patient satisfaction and adverse events were variable and limited by inconsistent reporting, precluding definitive conclusions regarding patient preference and comparative safety profiles. Overall, both treatments appear to be effective and generally well tolerated, with differences primarily in the type rather than the frequency of adverse effects. Given the heterogeneity of included studies and limitations in outcome reporting, treatment decisions should be individualized, considering patient characteristics, treatment preferences, and clinician expertise. Future research should focus on well-designed RCTs with standardized outcome measures and improved reporting of patient-centered outcomes to enable more definitive comparisons.

## Appendices

Appendix A

**Table 3:** Search strategy per database CINAHL: Cumulative Index to Nursing and Allied Health Literature; CENTRAL: Cochrane Central Register of Controlled Trials

MEDLINE		
S1	XB (microneedle* OR dermaroll* OR dermapen OR “percutaneous collagen induction”)	6,209
S2	(MM "Percutaneous Collagen Induction")	12
S3	S1 OR S2	6,218
S4	XB (“chemical peel*” OR chemexfoliation OR glycolic OR salicylic OR “trichloroacetic acid” OR TCA OR Jessner)	54,600
S5	(MM "Chemexfoliation")	702
S6	S4 OR S5	54,749
S7	XB acne	25,231
S8	(MM "Acne Vulgaris+") OR (MM "Acne Keloid") OR (MM "Acne Conglobata")	11,737
S9	S7 OR S8	26,406
S10	XB scar*	264,395
S11	S9 AND S10	2,546
S12	S3 AND S6 AND S11	16
Embase		
#1	(microneedle* or dermaroll* or dermapen or "percutaneous collagen induction").mp. [mp=title, abstract, heading word, drug trade name, original title, device manufacturer, drug manufacturer, device trade name, keyword heading word, floating subheading word, candidate term word]	8,969
#2	exp *microneedling/ or exp *microneedling device/	685
#3	1 or 2	9,369
#4	("chemical peel*" or chemexfoliation or glycolic or salicylic or "trichloroacetic acid" or TCA or Jessner).mp. [mp=title, abstract, heading word, drug trade name, original title, device manufacturer, drug manufacturer, device trade name, keyword heading word, floating subheading word, candidate term word]	98,850
#5	exp *trichloroacetic acid/ or exp *glycolic acid/ or exp *salicylic acid/ or exp *chemexfoliation/	13,524
#6	4 or 5	98,850
#7	acne.mp. [mp=title, abstract, heading word, drug trade name, original title, device manufacturer, drug manufacturer, device trade name, keyword heading word, floating subheading word, candidate term word]	53,301
#8	exp *acne keloidalis/ or exp *acne vulgaris/ or exp *acne cystica/ or exp *acne conglobata/ or exp *acne fulminans/ or exp *acne excoriee/ or exp *acne/	20,150
#9	7 or 8	53,858
#10	scar*.mp. [mp=title, abstract, heading word, drug trade name, original title, device manufacturer, drug manufacturer, device trade name, keyword heading word, floating subheading word, candidate term word]	392,407
#11	9 and 10	5,488
#12	3 and 6 and 11	85
Scopus		
#1	TITLE-ABS-KEY ( microneedle* OR dermaroll* OR dermapen OR "percutaneous collagen induction" )	10,512
#2	TITLE-ABS-KEY ( "chemical peel*" OR chemexfoliation OR glycolic OR salicylic OR "trichloroacetic acid" OR TCA OR Jessner )	133,925
#3	TITLE-ABS-KEY ( acne AND scar* )	4,992
#4	( TITLE-ABS-KEY ( microneedle* OR dermaroll* OR dermapen OR "percutaneous collagen induction" ) ) AND ( TITLE-ABS-KEY ( "chemical peel*" OR chemexfoliation OR glycolic OR salicylic OR "trichloroacetic acid" OR TCA OR Jessner ) ) AND ( TITLE-ABS-KEY ( acne AND scar* ) )	46
CINAHL		
S1	XB (microneedle* OR dermaroll* OR dermapen OR “percutaneous collagen induction”)	304
S2	(MM "Percutaneous Collagen Induction")	56
S3	S1 OR S2	353
S4	XB (“chemical peel*” OR chemexfoliation OR glycolic OR salicylic OR “trichloroacetic acid” OR TCA OR Jessner)	2,443
S5	(MM "Chemexfoliation")	125
S6	S4 OR S5	2,508
S7	XB acne	4,819
S8	(MM "Acne Vulgaris+") OR (MM "Acne Keloid") OR (MM "Acne Conglobata")	3,008
S9	S7 OR S8	5,852
S10	XB scar*	41,783
S11	S9 AND S10	305
S12	S3 AND S6 AND S11	0
CENTRAL		
#1	microneedle* OR dermaroll* OR dermapen OR “percutaneous collagen induction”	632
#2	MeSH descriptor: [Percutaneous Collagen Induction] explode all trees	50
#3	#1 OR #2	632
#4	(chemical NEXT peel*) OR chemexfoliation OR glycolic OR salicylic OR “trichloroacetic acid” OR TCA OR Jessner	2,748
#5	MeSH descriptor: [Chemexfoliation] explode all trees	121
#6	MeSH descriptor: [Salicylic Acid] explode all trees	466
#7	MeSH descriptor: [Trichloroacetic Acid] explode all trees	104
#8	#4 OR #5 OR #6 OR #7	2,806
#9	acne	6,434
#10	MeSH descriptor: [Acne Vulgaris] explode all trees	1,959
#11	MeSH descriptor: [Acne Keloid] explode all trees	11
#12	#9 OR #10 OR #11	6,434
#13	scar*	22,309
#14	#12 AND #13	864
#15	#3 AND #8 AND #14	21
	CENTRAL	19

Appendix B

**Table 4:** Eligibility screening matrix (full-text studies only)

Author and year	Title	Population	Intervention	Comparison	Outcome	Study type	Decision	Reason for exclusion	Documented reason for exclusion
Abdel-Magiud et al., 2020 [45]	Effects of different therapeutic modalities for postacne scars on circulating collagen III	Yes	Yes	Yes	No	Yes	Exclude	Reported outcomes for respective groups were on the serum collagen level	Desired outcome not reported
Al-Hamamy et al., 2023 [49]	Procedural combination of TCA-CROSS and microneedling in the treatment of grade-III and IV acne scars	Yes	No	No	Yes	Yes	Exclude	No monotherapies	Combination therapy
Ali et al., 2019 [25]	Microneedling (Dermapen) and Jessner's solution peeling in treatment of atrophic acne scars: a comparative randomized clinical study	Yes	Yes	Yes	Yes	Yes	Include	-	-
Anum et al., 2020 [46]	Outcome of microneedling combined with 35% focal trichloroacetic acid peeling in post acne atrophic scars	Yes	No	No	Yes	Yes	Exclude	The study combines microneedling and chemical peel	Combination therapy
Chandrashekar et al., 2014 [50]	Evaluation of microneedling fractional radiofrequency device for treatment of acne scars	Yes	No	No	Yes	No	Exclude	A radiofrequency device was used alone, not what we are looking for	Ineligible intervention
Deepika et al., 2024 [51]	The efficacy of the combination of microneedling with Jessner's Plus 35% trichloroacetic acid peel versus the efficacy of the combination of microneedling with platelet-rich plasma therapy for the treatment of atrophic acne scars: a comparative study	Yes	No	No	Yes	Yes	Exclude	No monotherapies	Combination therapy
El-Domyati et al., 2018 [44]	Microneedling combined with platelet-rich plasma or trichloroacetic acid peeling for management of acne scarring: a split-face clinical and histologic comparison	Yes	No	Yes	Yes	Yes	Exclude	Intervention was in combination	Combination therapy
Gadkari amd Nayak, 2014 [52]	A split-face comparative study to evaluate efficacy of combined subcision and dermaroller against combined subcision and cryoroller in treatment of acne scars	Yes	No	No	No	Yes	Exclude	Excluded as the intervention and comparator were not monotherapy but rather in combination	Combination therapy
Garg and Baveja, 2014 [53]	Combination therapy in the management of atrophic acne scars	Yes	No	No	Yes	Yes	Exclude	Combination research, no monotherapies	Combination therapy
Ishfaq et al., 2022 [28]	A comparison of microneedling versus glycolic acid chemical peel for the treatment of acne scarring	Yes	Yes	Yes	Yes	Yes	Include	-	-
Kondapally and Ashwani, 2023 [47]	Efficacy of subcision alone and with 50% TCA cross and microneedling in the management of atrophic acne scars	Yes	No	No	Yes	Yes	Exclude	The intervention and comparator weren't monotherapies	Combination therapy
Leelavathy et al., 2021 [54]	Efficacy and outcome of microneedling (Dermaroller) in post-acne scars	Yes	Yes	No	Yes	Yes	Exclude	There was no comparator	No comparator
Leheta et al., 2014 [55]	Do combined alternating sessions of 1540 nm nonablative fractional laser and percutaneous collagen induction with trichloroacetic acid 20% show better results than each individual modality in the treatment of atrophic acne scars? A randomized controlled trial	Yes	No	No	Yes	Yes	Exclude	Intervention was in combination. Comparator was a laser	Combination therapy
Leheta et al., 2014 [56]	Deep peeling using phenol versus percutaneous collagen induction combined with trichloroacetic acid 20% in atrophic post-acne scars; a randomized controlled trial	Yes	Yes	No	Yes	Yes	Exclude	Comparator was in combination with something else and not monotherapy	Combination therapy
Leheta et al., 2011 [22]	Percutaneous collagen induction versus full-concentration trichloroacetic acid in the treatment of atrophic acne scars	Yes	Yes	Yes	Yes	Yes	Include	-	-
Memon et al., 2022 [31]	Comparison of microneedling and glycolic acid peels in the management acne scar	Yes	Yes	Yes	Yes	Yes	Include	-	-
Memon et al., 2022 [29]	The comparison of treatment efficacy and outcomes of microneedling and glycolic acid peels for acne scar	Yes	Yes	Yes	Yes	Yes	Include	-	-
Minutilli, 2024 [57]	Microneedling for treatment of acne scars: considerations on the successful management of this aesthetic procedure	Yes	Yes	No	Yes	Yes	Exclude	There was no comparator; only microneedling was used	No comparator
Mohamed et al., 2022 [58]	Microbotox (Mesobotox) versus microneedling as a new therapeutic modality in the treatment of atrophic post‐acne scars	Yes	Yes	No	Yes	Yes	Exclude	Although microneedling was used in one study group, the other study group did not use chemical peels but rather Microbotox	Ineligible comparator
Mohta et al., 2024 [59]	Microneedling with autologous platelet-rich plasma versus topical insulin for treating postacne scars: a split-face comparison	Yes	No	No	Yes	Yes	Exclude	Combination study, excluding monotherapies	Combination therapy
Pakla-Misiur et al., 2021 [27]	Double-blind, randomized controlled trial comparing the use of microneedling alone vs chemical peeling alone vs a combination of microneedling and chemical peeling in the treatment of atrophic post-acne scars. An assessment of clinical effectiveness and patients' quality-of-life	Yes	Yes	Yes	Yes	Yes	Include	-	-
Puri, 2015 [23]	Comparative study of dermaroller therapy versus trichloroacetic acid CROSS for the treatment of atrophic acne scars	Yes	Yes	Yes	Yes	Yes	Include	-	-
Rana et al., 2017 [43]	Efficacy of microneedling with 70% glycolic acid peel vs microneedling alone in treatment of atrophic acne scars-a randomized controlled trial	Yes	No	Yes	Yes	Yes	Exclude	Intervention was not monotherapy	Combination therapy
Saadawi et al., 2019 [24]	Microneedling by dermapen and glycolic acid peel for the treatment of acne scars: comparative study	Yes	Yes	Yes	Yes	Yes	Include	-	-
Shahbano et al., 2023 [48]	Comparison of effectiveness and safety of 35% glycolic acid peel with microneedling versus glycolic acid peel alone in the management of acne scars	Yes	No	Yes	Yes	Yes	Exclude	Intervention was combined	Combination therapy
Sheraz et al., 2021 [30]	Efficacy of dermaroller vs trichloroacetic acid (TCA) cross technique for treatment of acne scars	Yes	Yes	Yes	Yes	Yes	Include	-	-
Srivastava and Garg, 2024 [21]	A comparative study of Dermaroller and salicylic acid peel in treatment of atrophic acne scars	Yes	Yes	Yes	Yes	Yes	Include	-	-
Ulfat et al., 2023 [60]	To compare the efficacy of 35% glycolic acid along with micro-needling versus platelet rich plasma along with micro-needling in treatment of the acne scars	Yes	No	No	Yes	Yes	Exclude	Comparator and intervention were combined with something else	Combination therapy
Ullah et al., 2020 [26]	Efficacy and safety of cross technique with 100% tca and dermaroller technique in the treatment of post acne scars	Yes	Yes	Yes	Yes	Yes	Include	-	-
Varma et al., 2018 [61]	Microneedling for atrophic post-acne scars: Is it effective? A prospective study of 36 cases at a tertiary care centre	Yes	Yes	No	Yes	Yes	Exclude	There was no comparator	No comparator

Appendix C

**Table 5:** Risk of bias decision details for randomized controlled trials NI: no information; Y: yes; N: no; PY: probably yes; PN: probably no; NA: not applicable

Unique ID	Ali et al., 2019 [25]	Study ID	Ali et al., 2019 [25]	Assessor	
Ref or label		Aim	Assignment to intervention (the "intention-to-treat" effect)		
Experimental		Comparator		Source	Journal article(s)
Outcome		Results		Weight	1
Domain	Signalling question			Response	Comments
Bias arising from the randomization process	1.1 Was the allocation sequence random?			NI	The allocation sequence was not mentioned
	1.2 Was the allocation sequence concealed until participants were enrolled and assigned to interventions?			NI	
	1.3 Did baseline differences between intervention groups suggest a problem with the randomization process?			NI	
	Risk of bias judgement			Some concerns	
Bias due to deviations from intended interventions	2.1 Were participants aware of their assigned intervention during the trial?			PY	
	2.2 Were carers and people delivering the interventions aware of participants' assigned intervention during the trial?			Y	
	2.3 If Y/PY/NI to 2.1 or 2.2: Were there deviations from the intended intervention that arose because of the experimental context?			NI	
	2.4 If Y/PY to 2.3: Were these deviations likely to have affected the outcome?			NA	
	2.5. If Y/PY/NI to 2.4: Were these deviations from intended intervention balanced between groups?			NA	
	2.6 Was an appropriate analysis used to estimate the effect of assignment to intervention?			PY	
	2.7 If N/PN/NI to 2.6: Was there potential for a substantial impact (on the result) of the failure to analyse participants in the group to which they were randomized?			NA	
	Risk of bias judgement			Some concerns	
Bias due to missing outcome data	3.1 Were data for this outcome available for all, or nearly all, participants randomized?			Y	
	3.2 If N/PN/NI to 3.1: Is there evidence that result was not biased by missing outcome data?			NA	
	3.3 If N/PN to 3.2: Could missingness in the outcome depend on its true value?			NA	
	3.4 If Y/PY/NI to 3.3: Is it likely that missingness in the outcome depended on its true value?			NA	
	Risk of bias judgement			Low	
Bias in measurement of the outcome	4.1 Was the method of measuring the outcome inappropriate?			N	
	4.2 Could measurement or ascertainment of the outcome have differed between intervention groups?			N	
	4.3 Were outcome assessors aware of the intervention received by study participants?			N	
	4.4 If Y/PY/NI to 4.3: Could assessment of the outcome have been influenced by knowledge of intervention received?			NA	
	4.5 If Y/PY/NI to 4.4: Is it likely that assessment of the outcome was influenced by knowledge of intervention received?			NA	
	Risk of bias judgement			Low	
Bias in selection of the reported result	5.1 Were the data that produced this result analysed in accordance with a pre-specified analysis plan that was finalized before unblinded outcome data were available for analysis?			Y	
	5.2 ... multiple eligible outcome measurements (e.g., scales, definitions, time points) within the outcome domain?			N	
	5.3 ... multiple eligible analyses of the data?			N	
	Risk of bias judgement			Low	
Overall bias	Risk of bias judgement			Some concerns	
